# Function of the Distal Part of the Vastus Medialis Muscle as a Generator of Knee Extension Twitch Torque

**DOI:** 10.3390/jfmk5040098

**Published:** 2020-12-18

**Authors:** Yoshitsugu Tanino, Takaki Yoshida, Wataru Yamazaki, Yuki Fukumoto, Tetsuya Nakao, Toshiaki Suzuki

**Affiliations:** Clinical Physical Therapy Laboratory, Kansai University of Health Sciences, 2-11-1Wakaba, Kumatori, Sennan, Osaka 590-0482, Japan; t.yoshida@kansai.ac.jp (T.Y.); w.yamazaki@kansai.ac.jp (W.Y.); fukumoto@kansai.ac.jp (Y.F.); nakao@kansai.ac.jp (T.N.); suzuki@kansai.ac.jp (T.S.)

**Keywords:** electrical muscle stimulation, knee extension, muscle fatigue, twitch torque, vastus medialis

## Abstract

The distal part of the vastus medialis (VM) (VM obliquus: VMO) muscle acts as the medial stabilizer of the patella. However, it has been known to facilitate VMO contraction during training of the quadriceps femoris muscle in knee joint rehabilitation. This study aimed to examine the contribution degree of VMO as a knee joint extension torque generator. Sixteen healthy male volunteers participated in this study. Electrical muscle stimulation (EMS) was performed on VMO at 60° knee angle for 20 min to induce muscle fatigue. Knee extension twitch torques (TT) at 90° and 30° knee angle evoked by femoral nerve stimulation were measured before and after EMS. Although each TT at 90° and 30° knee angle significantly decreased after EMS, the decreased TT rate in both joint angles showed no significant difference. Our results show that VMO might contribute to the generation of the knee joint torque at the same level in the range from flexion to extension. Therefore, it was suggested that the facilitating the neural drive for VMO is important during the quadriceps femoris muscle strengthening exercise.

## 1. Introduction

Torque generated at the knee through contraction of the quadriceps femoris muscle is essential for locomotion in humans. This torque affects the functional convalescence of patients with knee joint disease such as knee osteoarthritis and knee cruciate ligament injuries [1,2]. Therefore, the tension of the quadriceps femoris muscle should be transmitted to the tibial tuberosity to efficiently generate knee joint extension torque. The patella provides a component force to pull tibial tuberosity forward by bringing an angle of tilt to the patellar tendon in the sagittal plane in addition to lengthening the moment arm of the quadriceps femoris muscle and promotes efficiency of the torque occurrence [3]. However, due to the traction angle known as the Q-angle in the quadriceps femoris muscle, “laterally-directed force” acts on the patella during the muscle contraction [4]. The tension of the distal part of vastus medialis (VM obliquus: VMO) is known to stabilize the patella and efficiently generate knee joint extension torque [4,5,6]. Based on a muscle fiber orientation, VMO is not primarily involved in knee joint extension [5,7], but is thought to be the source that indirectly contributes to the knee joint extension torque, as mentioned above. Many therapists also believe that VMO contraction is an important function in knee rehabilitation [8]. However, the actual contribution of VMO in generating knee joint extension torque remains unclear. To investigate this, we tried to selectively induce the muscle fatigue of VMO for noninvasive in vivo examination because muscle fatigue is defined as a condition of inability to generate the expected force [9]. Therefore, we used electrical muscle stimulation (EMS) to induce VMO fatigue in this study and knee joint extension torque was investigated before and after the fatigue task. With regard to knee joint torque, the twitch torque (TT) evoked by femoral nerve stimulation was used as an index because the activity pattern of the quadriceps femoris muscle is different among participants under voluntary contraction. This study aimed to examine the contribution degree of VMO as a knee joint extension torque generator.

## 2. Materials and Methods

### 2.1. Participants

Sixteen healthy male volunteers without a history of knee joint injuries participated in this study. Their mean and standard deviations (SD) of age, height, and weight were 20.7 ± 0.6 years, 172.3 ± 5.9 cm, and 61.2 ± 6.2 kg, respectively. We sufficiently explained the purpose, method, risk, and renunciation upon their participation; written informed consent was obtained from all participants. This study was approved by the Research Ethic Review Committee of Kansai University of Health Sciences (approval number: 18–41, approval date: 16 February 2019).

### 2.2. Measurement of Knee Extension Torque

**Figure 1:**
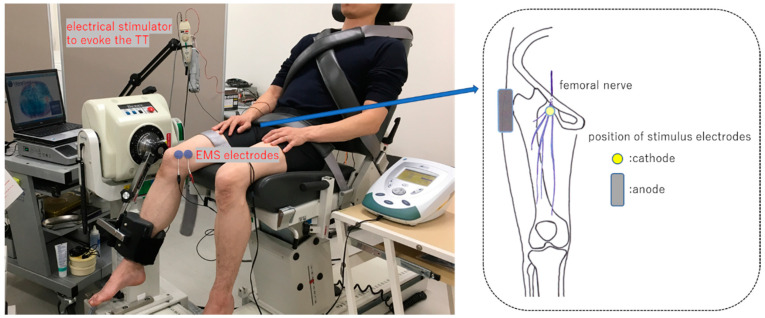
Experimental setup. Left picture shows the position of electrical muscle stimulation (EMS) electrodes on the vastus medialis obliquus muscle and electrical stimulator to evoke the twitch torque (TT). The position of stimulus electrodes connected to an electrical stimulator device is shown in the right figure.

Knee joint extension torques were measured from the dominant leg using the Biodex System 3 isokinetic dynamometer (Biodex Medical Systems Inc., Shirley, NY, USA). In addition, the leg used to kick a ball was considered as the dominant side. The measurement procedure for the knee joint torque in the isokinetic dynamometer was followed, matched with the participants’ knee joint and dynamometer axes, and regulated using a lever arm length of attachment so that a pad could fit the distal leg in the sitting position. The inclination angle of the backrest was adjusted so that the flexion angle of the hip joint was 60° (0° indicating neutral position of flexion/extension) (Figure 1).

The femoral nerve was percutaneously stimulated at the Scarpa’s triangle to evoke TT using an electrical stimulator device, i.e., electromyogram machine Viking Quest version 9.0 (Natus Neurology Inc., Middleton, WI, USA). A self-adhesive-type disposable disk electrode (20 mm diameter) was used as the cathode of stimulus electrode and was attached to the femoral nerve through the femoral artery pulse palpation in the Scarpa’s triangle. Then, a square self-adhesive-type disposable electrode (40 × 50 mm) was used as an anode and attached to the greater trochanter of the femur percutaneously (Figure 1). Electrical stimulus was defined as a rectangular wave of 0.5 ms duration with 120% intensity of the stimulation, requiring the maximum TT (supramaximal stimulation). TT was measured three times at 90° and 30° of the knee flexion angle (knee full extension, 0°), respectively, before and after a muscle fatigue task of the VMO described below and then, TT measurement of the knee joint angle was randomly performed. Data of TT output from the Biodex System 3 were recorded in the VitalRecorder2 waveform data collecting program (KISSEI COMTEC Co., Ltd., Nagano, Japan) by sampling 1000 Hz. Then, the TT curve was smoothed by a low-path filter of 6 Hz using the electromyogram analysis program BIMUTASU-Video (KISSEI COMTEC Co., Ltd., Nagano, Japan) and the peak value was calculated. The peak value of TT was normalized according to the participant’s body weight (Nm/kg).

### 2.3. Fatigue Task

EMS was conducted using the Intelect Mobile Stim (DJO Global LLC., Dallas, TX, USA) at 60° knee flexion angle. Two circular self-adhesive-type electrodes (32 mm diameter) were attached along a muscle fiber of VM inserted to the medial edge at the base of the patella for bipolar electrical stimulation (Figure 1). The ultrasonographic apparatus SONIMAGE HS1 (Konica Minolta Japan Inc., Tokyo, Japan) was used to identify the muscle fiber orientation, according to a previous study [7]. An investigator placed the probe of the ultrasonographic apparatus on the medial edge at the base of the patella, then turned a probe, and depicted a long axis image on the monitor so that the muscle fiber of VMO became straight. Next, two points at the top and bottom of the probe long axis were set on the skin with an oiliness pen; two EMS electrodes were attached so that the line connected these two points. Furthermore, this muscle fiber orientation was expressed at the angle to cross the long axis of the femur (a line that links the middle point at the base of the patella and anterior superior iliac spine) in the frontal plane and this angle was measured using the plastic goniometer. The edge of both the electrodes did not come in contact (interval, 2–3 mm) and these were attached to the muscle belly of the VMO. A symmetric bipolar wave (wave space, 0.1 ms) was used in the EMS task. The duration and frequency were 0.2 ms and 30 Hz, respectively. The stimulus intensity was decided by the participants using the indicated 7–8 numerical rating scale (NRS) after confirming an adequate muscle contraction of VM under both electrodes according to the investigator’s views. In addition, the participants’ intensities were measured every 5 min and electricity was consecutively turned on for 20 min while maintaining 7–8 NRS.

### 2.4. Statistical Analysis

The normality of TT peak value measured at each knee joint angle before and after EMS was confirmed using the Shapiro–Wilk test. Consequently, the TT peak value at each joint angle before and after EMS was compared using the paired t-test. Furthermore, the decreased TT rate (% value = 100–peak TT value after EMS/peak TT value before EMS × 100) was compared using the paired t-test. In addition, SPSS version 19 was used for the statistical analysis, with a significance level of 5%.

## 3. Results

The mean and SD of the VM muscle fiber orientation were 36.6° ± 2.7°. TT recorded at 90° was higher than that recorded at 30° knee flexion angle. Although each TT at 90° and 30° flexion angle significantly decreased after EMS to VM (Table 1), the decreased TT rate in both joint angles showed no significant differences (90°: 8.4% ± 7.7%; 30°: 9.9% ± 8.2%).

## 4. Discussion

The joint torque used to generate human movement is approximately decided according to the product of the moment arm and tension due to muscle contraction. The tension is influenced by the physiological cross-sectional area of the muscle, muscle length, and motor unit recruitment [10,11,12]. The factor of motor unit recruitment is thought to be uninvolved because the supramaximal stimulation to evoke the TT of knee extension can stably excite all axons of α-motoneurons in the femoral nerve, consequently contracting all muscle fibers of the quadriceps femoris muscle.

The tilting angle of the patellar tendon in addition to muscle length and moment arm is different in both 90° and 30° knee flexion angles. The moment arm is shorter at 90° knee flexion angle than at 30° knee flexion angle and the anterior drawer force for tibial tuberosity also becomes small due to decreased anterior tilting angle of the patellar tendon [3,13]. However, TT at 90° knee flexion angle was considered to be higher than that at 30° knee flexion angle due to lengthened quadriceps femoris muscle.

Muscle fatigue is defined as a condition of inability to generate the expected force and all factors in conjunction with the voluntary muscle contraction from the brain to muscle are involved in generating muscle fatigue [9]. Therefore, we have to measure the knee joint torque using the most suitable method to evaluate the influence due to VMO fatigue in the situation to not depend on the motor unit recruitment. Based on the result of this study, muscle fatigue of VMO was believed to be elicited because TT at each knee angle was significantly decreased after EMS to VMO and the decreased TT rate at 30° knee flexion angle tended to be higher than that at 90° knee flexion angle.

At 90° knee flexion angle, the patella is stabilized by setting the patellar groove of the femur [14]. Therefore, the VMO tension should counteract with the patellar lateral deviation during quadriceps contraction at 30° knee flexion angle compared with 90° knee flexion angle due to decreased patellar stability. Moreover, we speculated that the TT rate at 30° knee flexion angle is markedly decreased because the quadriceps tension does not efficiently transmit to the tibial tuberosity due to unstable patella during VMO fatigue. However, VMO was considered to have no knee extension action that contributes to TT generation at a similar level at both knee flexion angles.

VMO can pull the patella medially during the quadriceps contraction because this muscle attaches to the medial margin of the patella. VMO attaches to approximately 57% of ranges from the base of the patella and the fiber angle is approximately 56° against the femoral axis in healthy male participants [7]. Furthermore, the muscle fiber orientation becomes more horizontal (67.8° ± 4.0°) in high level competition athletes and it is thought that VMO contributes to the medial stabilization of the patella under the condition that the strong quadriceps femoris muscle contraction is required [15]. A muscle fiber angle of VM in this study was 36.6° ± 2.7°, which is low compared with the aforementioned reports [7]. The different points with this report were as follows; (1) knee joint angles were different when measuring muscle fiber angle (knee 0° in the aforementioned reports vs. knee 60° in this study), (2) it was a difference that measures a fiber angle attached to the most distal part of the patellar medial margin. The decreased TT rate in the knee flexion angle of 90° and 30° might yield a different result by reducing the VMO contraction ability that is attached to the medial margin of the patella. However, the area that can cause muscle fatigue by EMS is limited. Using the small EMS electrodes, the target muscle fibers might be contracted selectively. However, pain may easily occur during EMS using small electrodes because the current density becomes higher in small electrodes. Consequently, eliciting sufficient muscle contraction is difficult. In addition, it was considered as a method used to administer EMS to the motor point of VMO; however, contracting all muscle fibers in motor point stimulation is difficult [16]. Therefore, a method that ensures contraction of the area between two electrodes by bipolar electrical stimulation was used in this study and EMS is thought to be administered to the VM fiber, which attaches the medial margin of the patella at 60° knee flexion angle. However, the contractile ability of the VM longus (VML) was considered, which had knee joint extension action that might partially decrease, as fibers from the medial edge at the base of the patella were mainly contracted. VML partially attaches to the base of patella [17]. In addition, the fibers of VML have been reported to be strongly connected with the vastus intermedius (VI) muscle [18]. The VI has been reported to greatly contribute to the knee extension in the experiment using the knee in an above-knee amputation and electromyographic study [5,19]. Moreover, VI tension is considered to be easily transmitted to the bone directly [20]. The knee extension torque is efficiently generated by the VI contraction because the VML contraction increases VI tension [18]. As mentioned above, TT might decrease at almost the same level, regardless of the knee joint angle.

With regard to the limitations of this study, the knee extension torque was evaluated using the twitch in this study, although voluntary contraction is tetanic contraction. However, twitch summation through supramaximal stimulation inflicts significant pain in participants. Also, we could not evaluate the TT in the knee extended position because TT was difficult to evoke in this range (<30°).

## 5. Conclusions

The decreased TT rate was not significantly different at the two differential knee joint angles, although the contractile ability of VMO was decreased by muscle fatigue. Therefore, VMO might contribute to the generation of the knee joint torque at the same level in the range from flexion to extension. Based on this study, it was suggested that the facilitating the neural drive for VMO is important during the quadriceps femoris muscle strengthening exercise.

## Figures and Tables

**Table 1:** Each TT peak value (Nm/kg) at 90° and 30° knee flexion angle.

	Before EMS	After EMS
90° flexed position	0.50 ± 0.12	0.46 ± 0.12 *
30° flexed position	0.39 ± 0.10	0.35 ± 0.09 *

Data are expressed in mean ± SD. * Significant difference between before EMS and after EMS (*p* < 0.001).
